# Muscle Damage and Its Relationship with Muscle Fatigue During a Half-Iron Triathlon

**DOI:** 10.1371/journal.pone.0043280

**Published:** 2012-08-10

**Authors:** Juan Del Coso, Cristina González-Millán, Juan José Salinero, Javier Abián-Vicén, Lidón Soriano, Sergio Garde, Benito Pérez-González

**Affiliations:** Exercise Physiology Laboratory, Camilo José Cela University, Madrid, Spain; University of Cincinnati College of Medicine, United States of America

## Abstract

**Background:**

To investigate the cause/s of muscle fatigue experienced during a half-iron distance triathlon.

**Methodology/Principal Findings:**

We recruited 25 trained triathletes (36±7 yr; 75.1±9.8 kg) for the study. Before and just after the race, jump height and leg muscle power output were measured during a countermovement jump on a force platform to determine leg muscle fatigue. Body weight, handgrip maximal force and blood and urine samples were also obtained before and after the race. Blood myoglobin and creatine kinase concentrations were determined as markers of muscle damage.

**Results:**

Jump height (from 30.3±5.0 to 23.4±6.4 cm; *P*<0.05) and leg power output (from 25.6±2.9 to 20.7±4.6 W · kg^−1^; *P*<0.05) were significantly reduced after the race. However, handgrip maximal force was unaffected by the race (430±59 to 430±62 N). Mean dehydration after the race was 2.3±1.2% with high inter-individual variability in the responses. Blood myoglobin and creatine kinase concentration increased to 516±248 µg · L^−1^ and 442±204 U · L^−1^, respectively (*P*<0.05) after the race. Pre- to post-race jump change did not correlate with dehydration (r = 0.16; *P*>0.05) but significantly correlated with myoglobin concentration (r = 0.65; *P*<0.001) and creatine kinase concentration (r = 0.54; *P*<0.001).

**Conclusions/significance:**

During a half-iron distance triathlon, the capacity of leg muscles to produce force was notably diminished while arm muscle force output remained unaffected. Leg muscle fatigue was correlated with blood markers of muscle damage suggesting that muscle breakdown is one of the most relevant sources of muscle fatigue during a triathlon.

## Introduction

The triathlon is an endurance sport activity that combines phases of swimming, cycling and running. Although the most renowned distance for a triathlon race is the full distance (also known as Iron distance), shorter triathlon races (e.g. Olympic or half-iron distances) have become popular since they are more accessible to amateur and recreational athletes. Independent of the distance, the triathlon is one of the most fatiguing exercise activities since it combines long duration (from 1 h 50 min in the Olympic distance to more than 14 hours in the Ironman distance) with high exercise intensity [1]. However, only a small number of studies have been geared to determine the muscle fatigue experienced by triathletes. Margaritis et al. [2] found a decreased capacity to generate force in the knee extensor and flexor muscles after a half-iron triathlon. Similarly, Suzuky et al. [3] found that jump height during squat and countermovement jumps (CMJ) significantly decreased after an Iron triathlon along with a reduction in the maximal isometric strength of the knee extensors. Thus, it seems apparent that muscle performance is diminished after a triathlon race, but its origin remains elusive.

According to Jeukendrup [1], dehydration and carbohydrate depletion are the most likely causes of fatigue during a triathlon race. Dehydration alone [4] or in combination with hyperthermia [5], [6] may lessen maximal isometric strength production. Thus, drinking during exercise to avoid dehydration and hyperthermia should be a primary goal for triathletes, especially to avoid a body mass loss superior to 2% [7]. However, it has been found that triathletes do not dehydrate excessively and that the body mass loss experienced during the triathlon is mostly due to reduced fat and skeletal muscle mass [8], [9]. In addition, it has been found that the dehydration level does not correlate with total race time in a triple iron triathlon [10]. According to these studies, triathletes seem well informed about the necessity of rehydrating during exercise, diminishing the deleterious effects of dehydration during a triathlon.

During exercise, an insufficient supply of glucose may result in hypoglycemia and consequently muscle fatigue. For this reason, several investigations have established that the ingestion of carbohydrates improves endurance capacity by maintaining blood glucose content [11], [12]. Carbohydrate ingestion also helps to maintain muscle force production and central nervous system (CNS) activation during prolonged exercise [13]. In triathlon male athletes, it has been found that race finishing time was inversely related to carbohydrate intake during the race, although this relation was not found in females [14]. Since the rate of exogenous carbohydrate oxidation is close to1 g/min, a carbohydrate intake of ∼60 g/hour has been suggested [15]. Therefore, the intake of carbohydrates during a triathlon to preserve the homeostasis of blood glucose is a crucial factor for avoiding muscle fatigue.

A reduction in plasma sodium concentration below 135 mmol · L^−1^, mainly as a result of excessive fluid consumption, has been previously reported in triathletes [9], [16]. This electrolyte imbalance is considered as a serious medical problem during ultra-distance events [1] since it is associated with weakness and mental confusion. Even coma and death may occur with sodium concentrations below 126 mmol · L^-1^. However, the association between the decrease in plasma sodium content and muscle fatigue has not been previously established in triathletes. During prolonged cycling in the heat, Coso et al. [5] found that the ingestion of rehydrating drinks with low sodium content reduced plasma sodium concentration and worsened the maintenance of isometric muscle strength. This negative effect on muscle strength was present even when plasma sodium was not lower than 137 mmol · L^-1^, so the reduction in sodium concentration may affect muscle performance with values higher than those typically related with hyponatremia.

Another factor that could affect muscle fatigue in the triathlon is myofibril damage, mainly produced during the running leg. Swimming and cycling are activities that produce minor muscle damage in the involved muscles. However, running is a weight-bearing activity that includes concentric and eccentric actions in the leg muscles. In a recent study, it has been found that urinary myoglobin concentration (a marker of muscle breakdown) correlates with leg muscle power reduction after a marathon [17], although other blood markers of muscle damage do not correlate with muscle fatigue after a triathlon [2]. The purpose of this study was to investigate the cause/s of muscle fatigue which ensues during a half-iron distance triathlon. We hypothesized that the source of muscle fatigue will be multifactorial, with dehydration, hypoglycemia, electrolyte imbalance and muscle damage as the main factors responsible for muscle force loss during a half-iron triathlon.

## Methods

### Subjects

Thirty one triathletes volunteered to participate in this investigation. However, six of these participants failed to complete the triathlon race and their records were excluded from the study. Thus, this investigation includes data from 25 healthy and well-trained triathletes. All the participants had previous experience of at least 3 yrs and had trained for ∼2 h · day^−1^, 4–5 days · week-1 during the previous year. In addition, the participants had completed at least one prior triathlon in the half-iron distance. The characteristics of the participants are summarized in Table 1. Participants completed a short questionnaire on training status and medical history. Potential participants with a history of muscle disorder, cardiac or kidney disease or those taking medications were excluded.

**Table 1:** Morphological characteristics of the participants and their performance in the Half-Ironman triathlon race. Data are mean±SD and ranges for 25 healthy triathletes.

	Mean ± SD	Range
Age (yr)	35.7±6.5	25−54
Height (cm)	178±7	160−191
Weight (kg)	75.1±9.8	58.4−92.5
Body fat (%)	8.6±3.9	3.4−16.0
Half-iron triathlons completed	17±22	1−97
Total race time	5:12:20±00:34:59	04:05:55−06:08:06
2.0 km swim time	00:33:52−00:04:11	00:27:13−00:42:33
90 km cycle time	02:44:53−00:18:37	02:11:16−03:17:07
Half-marathon time	01:49:01−00:27:23	01:26:43–02:19:53

### Ethics Statement

Participants were fully informed of any risks and discomforts associated with the experiments before giving their informed written consent to participate. The study was approved by the Camilo Jose Cela Ethics Committee in accordance with the latest version of the Declaration of Helsinki.

### Experimental Protocol

One to three days before the race, participants underwent a physical examination to ensure that they were in good health. Ninety-to-sixty minutes before the race, participants arrived at a zone close to the start line to assess pre-exercise variables. Participants voided and a urine sample was obtained to determine urine specific gravity (U_sg_) and other urinary variables. Participants' body fat composition and pre-race body water content were then calculated using bioimpedance (BC-418, Tanita, Japan). After that, a 22-G catheter was inserted into an antecubital vein and a 7 mL blood sample was drawn. Next, participants completed a 10 min warm-up consisting of dynamic exercises and practice jumps. After that, participants performed two countermovement vertical jumps for maximal height on a force platform (Quattrojump, Kistler, Switzerland). Handgrip maximal force production in both hands was measured by using a handgrip dynamometer (Grip-D, Takei, Japan). Finally, participants were weighed in their competition clothes (±50 g scale; Radwag, Poland) and headed to the start line to participate in a half-iron distance triathlon.

The race consisted of 1.9 km of swimming, 75 km of cycling (1100 m of net increase in altitude) and 21.1 km of running. The triathlon was held in June 2011 in the surrounding area of a city located at 975 m altitude. Mean±SD (range) dry temperature during the event was 22.3±6.9°C (13−30°C) with a relative humidity of 72.8±8.0% (65−85%). Water temperature during the swim section was 19±1°C. No instructions about pace, drinking or feeding during the race were given to the participants to avoid any influence of this investigation on their habitual routines during the race. So, participants drank and consumed food *ad libitum* and swam, cycled and ran at their own pace. Performance times for the swimming, cycling and running phases and the total time are shown in Table 1.

Within 3 min of the end of the race, participants went to a finish area and performed two countermovement vertical jumps (see below). Post-race body weight and body water content were then recorded with the same devices and clothes used for the pre-race measurement. Participants were instructed to avoid drinking from the finish line till the post-race weighing and an experimenter assured compliance. Then, participants rested for five minutes and a venous blood sample was obtained using the procedures described previously. After that, subjects were provided with fluid (water and sports drinks) to promote urine production. Thirty to 60 minutes after the race, a representative sample of the first post-race void was collected in a sterile container. After that, participants finished their participation in the study. The dehydration level attained during the race was calculated as percent reduction in body weight (pre-to post-race), assuming that body mass changes was produced by a reduction in participants' water content. Similarly, we calculated the reduction in body water content using pre and post-race body water measurements by bioimpedance.

### Maximal countermovement jump

Before and just after the race, participants performed two maximal countermovement jumps on a force platform (Quattrojump, Kistler, Switzerland) to assess changes in jump height and leg power production. For this measurement, participants began stationary in an upright position with their weight evenly distributed over both feet. Each participant placed their hands on their waist in order to remove the influence of the arms on the jump. On command, the participant flexed their knees and jumped as high as possible while maintaining the hands on the waist and landed with both feet. After 1 min rest, the countermovement jump was repeated. Leg muscle power output during the impulse phase (concentric part of the jump) and jump height were determined as previously described [17].

**Figure 1:**
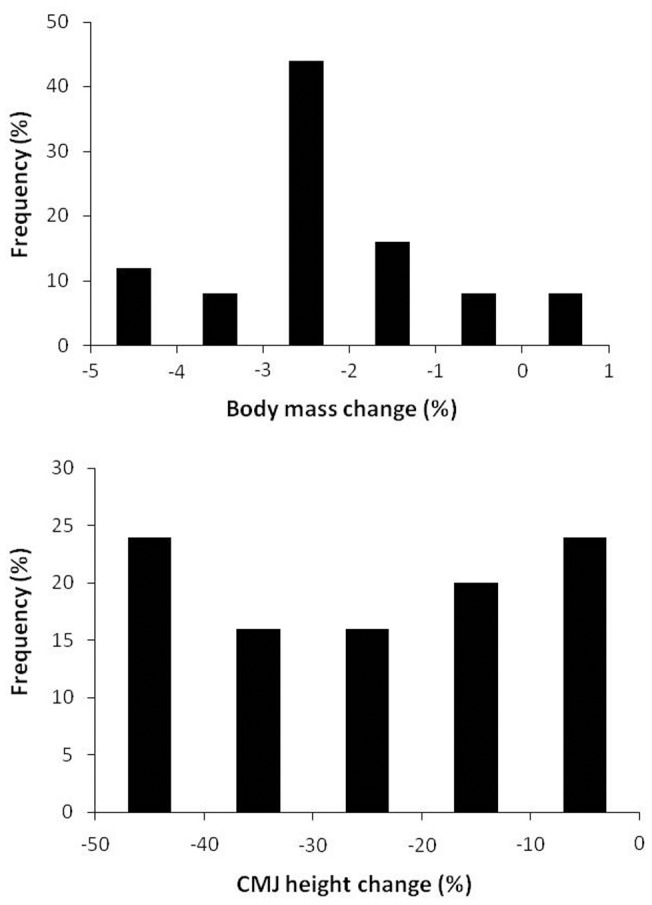
Body mass and jump height changes after a half-iron distance triathlon. Data are frequencies for 25 experienced triathletes.

**Figure 2:**
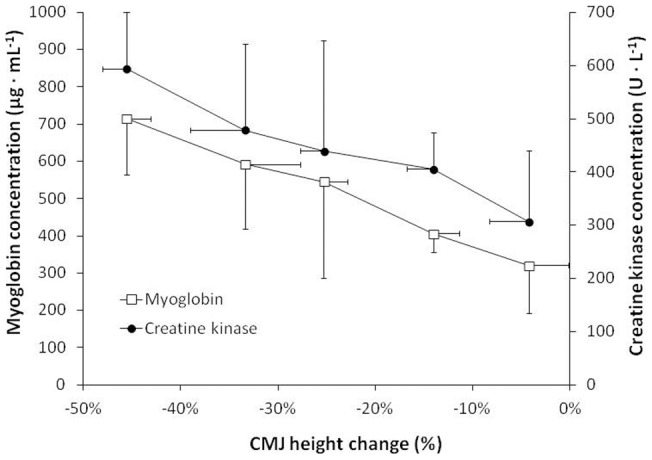
Associations among jump height loss and blood markers of muscle damage. Relationship between the changes in jump height (x-axis) and serum myoglobin concentration (y-axis 1) and serum creatine kinase concentration (y-axis 2) after a half-iron distance triathlon. Data mean ± SD for 25 experienced triathletes grouped by their jump height loss after race.

### Blood samples

A portion of each blood sample (2 mL) was introduced into a tube with EDTA and blood glucose concentration (Accu-chek, Spain), hemoglobin concentration and hematocrit were determined within the same day. Changes in blood volume and plasma volume from rest were calculated with the equations outlined by Dill and Costill [18]. Red blood cell, white blood cell and platelet counts were determined by using an automated blood counter. The remaining blood (5 mL) was allowed to clot and serum was separated by centrifugation (10 min at 5000 g) and frozen at −80°C until the day of analysis. On a later day, the serum portion was analyzed for sodium, potassium and chloride concentrations (Nova 16, NovaBiomedical, Spain). In addition, myoglobin, creatine kinase (CK) and lactate dehydrogenase (LDH) concentrations were measured as blood markers of muscle damage.

### Urine samples

Pre and post-race urine samples were immediately analyzed (within 2 hours) for specific gravity (Usg), pH, protein, glucose, ketones and bilirubin concentrations. We also determined the presence of leukocytes and erythrocytes in the urine using reactive strips (Combur Test, Roche, Switzerland). For these measurements, the strip was dipped in the urine sample and the excess was wiped off with a clean absorbent paper. Then, the test strip was placed on the tray of a photometer (Urisys 1100, Roche Switzerland) and the aforementioned variables were measured after 1 min of incubation. After each ten sample batch, the photometer was calibrated with control strips provided by the manufacturer.

### Statistical Analysis

Data are presented as mean ± SD. Initially, we tested the normality of each variable with the Shapiro-Wilk test. Changes in the variables from pre to post-race were analyzed with Student's t test for paired samples. To simplify the presentation of data, participants were grouped by their change in jump height using 10% intervals. Urine variables were presented by the frequency of subjects that presented a determined value. We used Pearson's correlation to assess the association between two variables. The significance level was set at *P*<0.05. We also performed a multiple regression analysis in a stepwise interactive mode, based on previous investigations [19] aiming to assess the influence of the measured variables on the muscle fatigue experienced during a half-iron triathlon (i.e., CMJ height loss).

For this calculation, the measured variables in the study were included based on their correlation with the residual (*P*<0.1) and their intercorrelation with variables already in the equation. The regression equation produced was accepted at a significance level of *P*<0.01. The degree of variance on CMJ height loss explained by means of each parameter was calculated using regression coefficients. Using the standardized regression coefficients, the relative contribution of the different variables to the variance explained was calculated as follows:
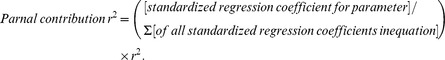



Finally, the r^2^values were adjusted for the number of cases and the number of parameters in the analysis. This statistical analysis was performed using the SPSS v.18 software package (SPSS Inc., USA).

## Results

### Dehydration and body water change

After the race, most participants had reduced their pre-exercise body mass (from 75.2±9.6 to 73.3±9.5 kg; *P*<0.05) with a mean dehydration of 2.3±1.2%. However, the dehydration attained after the race was diverse among individuals (Figure 1A). Most participants (44% of the total) reduced their body mass between 2 and 3% while only 12% of participants dehydrated more than 4% (peak dehydration was 4.3%). On the contrary, 8% of the triathletes slightly increased their body weight with a maximal gain of 0.3%. Body water followed a similar pattern (from 48.8±5.4 kg before the race to 46.5±5.5 kg after the race; *P*<0.05) with a mean body water deficit of 4.2±2.1%. The dehydration level attained during the race and the body water deficit were significantly correlated (r = 0.71; *P*<0.05). However, there was no significant correlation between dehydration and CMJ height change (r = −0.16; *P* = 0.44) or body water loss and CMJ height change (r = −0.01; *P* = 0.96).

### Countermovement jump height and handgrip force

Before the race, mean CMJ jump height was 30.3±5.0 cm and mean power output during the concentric phase of the jump was 25.6±2.9 W kg^−1^. After the race, CMJ jump height (23.4±6.4 cm; *P*<0.05) and jump power output (20.7±4.6 W/kg; *P*<0.05) were significantly reduced by 23±16% and 19±15%, respectively. Although all participants reduced their CMJ jump from pre-exercise values, there was an elevated inter-individual variability in the responses (Figure 1B). On the contrary, handgrip maximal force production in the dominant (from 438±57 N pre-exercise to 436±65 N after the race; *P* = 0.85) and non-dominant hand (from 422 N±63 pre-exercise to 423±59 N after the race; *P* = 0.77) was unaffected after the triathlon race.

### Blood responses

From pre-race values, blood volume and plasma volume were significantly reduced by 2.9±3.0% and 4.3±3.0%, respectively (*P*<0.05). The changes in the remaining blood variables are shown in Table 2. As a consequence of plasma volume reduction, hemoglobin and hematocrit concentration increased after the race (*P*<0.05). Post-exercise platelet count increased by 15±9% (*P*<0.05), leukocyte count by 200±9% (*P*<0.05) while erythrocytes remained unchanged. Post-race blood glucose concentration increased by 15±5 mg · dL^−1^ in comparison to pre-race values (*P*<0.05). While sodium and chloride concentrations were significantly reduced after the race (*P*<0.05), and potassium concentration slightly increased (*P*<0.05). Finally, the concentrations of all the blood markers of muscle damage showed varying increases, from pre to post race (*P*<0.05). There was a negative correlation between the CMJ jump height change and post-race myoglobin concentration (r = −0.65; *P*<0.001) and post-race creatine kinase concentration (r = −0.54; *P*<0.001; Figure 2), but this correlation was not significant with the LDH concentration (r = 0.01; *P* = 0.96).

**Table 2:** Blood responses before (Pre) and after (Post) a half-iron triathlon race. Data are mean ± SD for 25 triathletes.

*Variable (units)*	Pre	Post	P value
Hemoglobin (g · dL^−1^)	14.7±0.8	15.2±0.9	<0.05
Hematocrit (%)	45.4±2.4	46.3±2.4	<0.05
Erythrocytes (10^9^ · L^−1^)	4926±310	4898±351	NS
Leukocytes (10^9^ · L^−1^)	5.5±1.0	16.1±2.5	<0.05
Platelets (10^9^ · L^−1^)	249±36	286±46	<±0.05
Glucose (mmol · L^−1^)	5.1±0.7	6.3±1.6	<0.05
Sodium (mmol · L^−1^)	141.4±1.6	140.4±1.9	<0.05
Potassium (mmol · L^−1^)	4.5±0.2	4.7±0.7	<0.05
Chloride (mmol · L^−1^)	101.2±2.0	98.3±2.4	<0.05
Myoglobin (µg · L^−1^)	14±17	516±248	<0.05
Creatine kinase (U · L^−1^)	145±72	442±204	<0.05
LDH (U · L^-1^)	298±66	598±252	<0.05

### Urinary responses

Before the race, all the twenty five triathletes had U_sg_ below 1.020. Although U_sg_ values significantly increased from pre-to post-race (Table 3; *P*<0.05), only 2 participants (8% of the sample) exceeded 1.020 after the race. The half-iron triathlon increased the urinary concentration of erythrocytes, leukocytes, proteins and ketones by varying amounts (*P*<0.05). On the contrary, urine pH and bilirubin concentration remained unchanged after the race.

## Discussion

**Figure 3:**
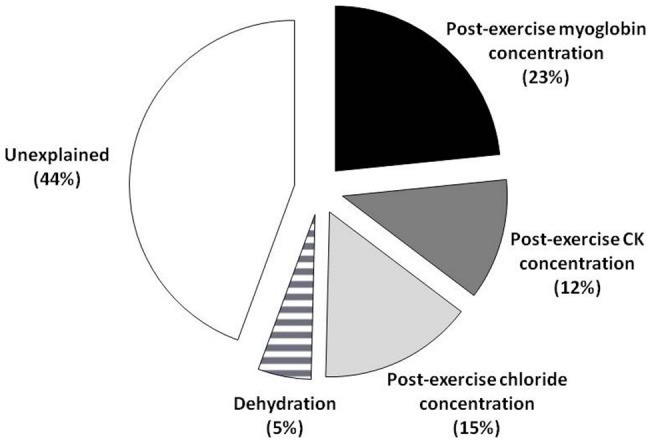
Amount of variance explained (adjusted r2) for CMJ height loss after the half-iron triathlon. The best regression equation includes blood variables (post-exercise myoglobin, creatine kinase and chloride concentrations) and the dehydration attained after the race. The remaining portion represents the unexplained variance.

The aim of this investigation was to determine the sources of muscle fatigue experienced by triathletes during a half-iron distance race. According to previous investigations, dehydration, hypoglycemia, electrolyte imbalance and muscle damage are probable factors affecting muscle force output in the triathlon. In the present investigation, we measured all these variables before and after a half-iron race and performed a multiple regression analysis to determine the influence of each variable on the pre-post race change in CMJ height. The main outcomes were: (a) body mass loss (Figure 1A) and body water deficit after the race were moderate and these variables were modestly correlated with jump height loss; (b) blood glucose content increased while serum sodium and chloride concentrations decreased after the race (Table 2), although their values were far from constituting a serious electrolyte imbalance; (c) blood markers of muscle damage strongly correlated with jump height loss (Figure 2), suggesting that muscle breakdown is one of the primary causes of muscle fatigue during a triathlon. With all these data, we have explained 56% of the variance in muscle fatigue which ensued during a half-iron triathlon (Figure 3).

**Table 3:** Urinary responses before (Pre) and after (Post) a half-iron triathlon race. Data are mean±SD for 25 triathletes.

*Variable (units)*	Pre	Post	P value
U_sg_	1.012±0.006	1.018±0.006	<0.05
pH	6.2±1.1	6.1±1.2	0.84
Erythrocytes (U · µL^−1^)	1.4±0.3	18.3±51.5	<0.05
Leukocytes (U · µL^−1^)	0.0±0.0	7.3±11.6	<0.05
Proteins (mg · dL^−1^)	3.0±3.3	89.6±134.9	<0.05
Ketones (mg · dL^−1^)	0.2±1.0	24.4±39.9	<0.05
Bilirubin (mg · dL^−1^)	0.2±0.6	0.2±0.4	0.71

All the triathletes that participated in this study experienced a reduction in jump height and leg power production after the race, a clear sign of muscle fatigue as found previously [3]. However, the individual responses for jump height change were diverse (Figure 1B). The explanation for the variance in muscle fatigue responses after the triathlon race is presented in Figure 3. Pre-to-post race change in jump height depended on the post-exercise blood myoglobin concentration (23%), the post-exercise blood chloride concentration (15%), the post-exercise blood CK concentration (12%) and dehydration (5%). With this model we can determine which factors modulate muscle fatigue during a triathlon race. Nonetheless, there is still a high proportion of muscle fatigue variability that remains unexplained with our experimental design, which warrants further investigation.

According to the multifactorial analysis, 35% of jump height loss experienced by the triathletes is explained by means of blood markers of muscle damage (i.e., myoglobin and CK). Although we did not measure CK isoforms (e.g. MM, BB and MB) to differentiate the origin of muscle breakdown, we assume that the largest part of the CK increase found in the post-race blood samples was from skeletal muscle. As presented in Figure 2, those triathletes with a higher reduction in jump height were the ones with a higher concentration of myoglobin and CK after the race. These data suggest that muscle breakdown produced in the triathlon is one of the primary factors for muscle fatigue in triathletes. While there is no study that has reported moderate-to-severe muscle damage after swimming or cycling, the references to muscle damage after endurance running are abundant [20], [21], [22]. The continuous foot strikes during the running leg demand concentric and eccentric actions of the leg muscles and can damage muscle fibers [23].

During an iron triathlon, Margaritis et al. [2] did not find an association between blood CK concentration and the reduction in knee extensor and flexor muscle force production during several days after the race. These authors concluded that blood markers of muscle damage cannot be used to predict the magnitude of the muscle function impairment during the triathlon. In contrast, Coso et al. [17] found a relationship between the myoglobin concentration in the first urine after a marathon race and leg muscle power reduction, concluding that muscle damage is a good predictor of leg muscle fatigue after running. The present investigation also indicates that muscle damage, estimated by blood myoglobin and CK concentrations, is the main factor affecting muscle fatigue in triathletes. Thus, the strategies to diminish the extent of muscle fatigue in triathletes should include the avoidance of muscle damage.

A dehydration level above 2% has been repeatedly related to muscle force loss during endurance exercise events [7] and it has been proposed as one of the main factors responsible for fatigue during triathlon events [1]. In the present study, however, the variance in CMJ height loss explained by means of dehydration was only 5% (Figure 3) which suggests that the influence of dehydration on muscle fatigue during the triathlon was minor. Although dehydration was present in most triathletes, the levels attained by the majority of triathletes were moderate (mean 2.3±1.2%). Thirty two percent of the sample dehydrated less than 2%, 52% of the participants dehydrated between 2 and 4% and the remaining 12% dehydrated more than 4% (Figure 1A). The poor association between dehydration and the loss of CMJ height indicates that the triathletes with higher levels of dehydration did not loss more jump height than the ones with better rehydration during the race. We assume that the proper drinking behavior showed by most triathletes in this study reduced the influence of dehydration on the muscle fatigue.

The concurrence of sodium loss by sweating and the high intake of water or low-sodium drinks during long distance triathlon causes some triathletes to present hyponatremia (<135 mmol · L^−1^; [9], [16]). This electrolyte imbalance has been related to clinical complications in exercise medicine, since its symptoms include weakness, fainting or even death. However, blood sodium concentration may also play an important role in the preservation of muscle function during exercise. Vrijens and Reher [24] reported a relationship between decreased blood sodium concentration and time to fatigue during a prolonged cycling event. Similarly, Coso et al. [5] found a relationship between sodium deficit and muscle fatigue during prolonged cycling even with a mean sodium concentration above 137 mmol · L^−1^. In the present investigation, blood sodium concentration decreased from 141 to 140 mmol · L^−1^ but this variable was not correlated with CMJ height loss (r = 0.02; *P* = 0.92). In contrast, blood chloride content changed from 101 to 98 mmol · L^−1^ and post-exercise values significantly correlated with CMJ height loss (r = 0.34; *P*<0.05). It has been found that intramuscular sodium and chloride contents remain unchanged after prolonged exercise that produces profuse sweating while the excitability of the muscle cell membrane is unaffected. Although lacking a clear explanation, maintaining blood electrolyte concentration during exercise may help to preserve muscle function.

During exercise of ∼1 hour of duration, glucose supply for the skeletal muscle comes from glycogen stores in the muscle and liver. If the exercise bout is of long duration (>1 hour), main glycogen stores deplete and blood borne glucose is also used as the energy substrate, threatening blood glucose homeostasis [25]. It has been found that hypoglycemia attenuates the activation of the CNS and hence reduces the capacity to generate force in the active muscle [13]. For this reason, the reduction in blood glucose concentration has been proposed as a source of muscle fatigue during the triathlon [1]. When blood glucose is maintained by ingesting carbohydrates during exercise, muscle force and CNS activation are better preserved [13]. Interestingly, participants in this investigation increased by 0.8±0.5 mmol · L^−1^ the blood glucose concentration from pre-to-post exercise (Table 2), as has been previously found in other athletes participating in endurance events [26]. Although we did not record carbohydrate ingestion during the race, previous studies have found that triathletes have appropriate rates of carbohydrate intake, especially during the cycling leg [14] According to our data, blood glucose concentration was well maintained in triathletes, reducing the influence of hypoglycemia as a source of fatigue during a half-iron race.

Several studies have reported that muscle force production is ameliorated in hyperthermic individuals with or without dehydration [6], [27], [28]. During prolonged cycling in the heat that produced hyperthermia, Nybo and Nielsen [27] reported a reduction in both leg and arm force production, despite arm muscles not being involved during the cycling activity. These authors suggested that hyperthermia produces a central effect that affects muscle function in both active and non active muscles, although different outcomes have been found in another analogous study [29]. In the present investigation we did not assess body temperature, but during an iron triathlon with an environmental temperature similar to the present investigation (23.3 *vs* 22.3°C), mean core temperature in triathletes was only 38.1°C [30]. In addition, while leg muscle power and jump height were reduced after the race, handgrip force remained unchanged from pre-to-post exercise, contrary to the results reported by Nybo and Nielsen. The maintenance of arm force while leg force was reduced disagrees with the existence of hyperthermia and its effects on CNS activation.

Exercise, particularly high-intensity and endurance activities produce several urinary abnormalities. Hematuria is one of the most commonly found abnormalities after sports activity [31] and it is present with a higher frequency in weight-bearing exercise activities (running *vs* cycling; [32]). Others studies have reported that hematuria is present in 20-to-50% of marathon finishers [33], [34]. The present study found that erythrocyte concentration increased from 1.4±0.3 to 18.3±51.5 U · µL^-1^ in the first urine after the triathlon. In addition, the prevalence of hematuria increased from 0 to 36% after the race, with one participant above 250 U · µL^−1^. Although there was no incidence of kidney complications after the race, these data suggest the necessity of obtaining an exercise history when urinary abnormalities are present in triathletes.

In summary, during a half-iron triathlon in a temperate environment, leg muscle function was significantly impaired while muscle force in the upper extremities remained unchanged. Furthermore, the source of leg muscle fatigue experienced by triathletes was diverse and depended on several factors. Post-exercise myoglobin and creatine kinase concentrations correlated with CMJ height loss after the race, indicating that muscle fiber damage is one of the key factors for muscle fatigue in the triathlon. In contrast, dehydration and blood glucose concentration had a minor role in muscle fatigue mainly due to appropriate rehydrating and feeding during the race. Strategies to lessen muscle fatigue during triathlon events should comprise a reduction in muscle damage.

## References

[1] Jeukendrup AE, Jentjens RL, Moseley L (2005) Nutritional considerations in triathlon. Sports Med 35: 163–181. 15707379 10.2165/00007256-200535020-00005

[2] Margaritis I, Tessier F, Verdera F, Bermon S, Marconnet P (1999) Muscle enzyme release does not predict muscle function impairment after triathlon. J Sports Med Phys Fitness 39: 133–139. 10399422

[3] Suzuki K, Peake J, Nosaka K, Okutsu M, Abbiss CR, et al (2006) Changes in markers of muscle damage, inflammation and HSP70 after an Ironman Triathlon race. Eur J Appl Physiol 98: 525–534. 17031693 10.1007/s00421-006-0296-4

[4] Judelson DA, Maresh CM, Anderson JM, Armstrong LE, Casa DJ, et al (2007) Hydration and muscular performance: does fluid balance affect strength, power and high-intensity endurance? Sports Med 37: 907–921. 17887814 10.2165/00007256-200737100-00006

[5] Coso JD, Estevez E, Baquero RA, Mora-Rodriguez R (2008) Anaerobic performance when rehydrating with water or commercially available sports drinks during prolonged exercise in the heat. Appl Physiol Nutr Metab 33: 290–298. 18347684 10.1139/H07-188

[6] Coso JD, Hamouti N, Estevez E, Mora-Rodriguez R (2011) Reproducibility of two electrical stimulation techniques to assess neuromuscular fatigue. Eur J Sport Sci 11.

[7] Sawka MN, Burke LM, Eichner ER, Maughan RJ, Montain SJ, et al (2007) American College of Sports Medicine position stand. Exercise and fluid replacement. Med Sci Sports Exerc 39: 377–390. 17277604 10.1249/mss.0b013e31802ca597

[8] Knechtle B, Knechtle P, Rosemann T, Oliver S (2010) A Triple Iron triathlon leads to a decrease in total body mass but not to dehydration. Res Q Exerc Sport 81: 319–327. 20949852 10.1080/02701367.2010.10599680

[9] Speedy DB, Noakes TD, Kimber NE, Rogers IR, Thompson JM, et al (2001) Fluid balance during and after an ironman triathlon. Clin J Sport Med 11: 44–50. 11176145 10.1097/00042752-200101000-00008

[10] Knechtle B, Duff B, Amtmann G, Kohler G (2008) An ultratriathlon leads to a decrease of body fat and skeletal muscle mass–the Triple Iron Triathlon Austria 2006. Res Sports Med 16: 97–110. 18569944 10.1080/15438620701878881

[11] Jeukendrup A, Brouns F, Wagenmakers AJ, Saris WH (1997) Carbohydrate-electrolyte feedings improve 1 h time trial cycling performance. Int J Sports Med 18: 125–129. 9081269 10.1055/s-2007-972607

[12] Coggan AR, Coyle EF (1987) Reversal of fatigue during prolonged exercise by carbohydrate infusion or ingestion. J Appl Physiol 63: 2388–2395. 3325488 10.1152/jappl.1987.63.6.2388

[13] Nybo L (2003) CNS fatigue and prolonged exercise: effect of glucose supplementation. Med Sci Sports Exerc 35: 589–594. 12673141 10.1249/01.MSS.0000058433.85789.66

[14] Kimber NE, Ross JJ, Mason SL, Speedy DB (2002) Energy balance during an ironman triathlon in male and female triathletes. Int J Sport Nutr Exerc Metab 12: 47–62. 11993622 10.1123/ijsnem.12.1.47

[15] Jeukendrup AE, Jentjens R (2000) Oxidation of carbohydrate feedings during prolonged exercise: current thoughts, guidelines and directions for future research. Sports Med 29: 407–424. 10870867 10.2165/00007256-200029060-00004

[16] Speedy DB, Rogers IR, Noakes TD, Wright S, Thompson JM, et al (2000) Exercise-induced hyponatremia in ultradistance triathletes is caused by inappropriate fluid retention. Clin J Sport Med 10: 272–278. 11086754 10.1097/00042752-200010000-00009

[17] Coso JD, Salinero JJ, Abián-Vicen J, González-Millán C, Garde S, et al.. (2012) Dehydration or rhabdomyolysis to predict muscle fatigue during a marathon in the heat? Annual conferece of the Spanish Olympic Committee In press.

[18] Dill DB, Costill DL (1974) Calculation of percentage changes in volumes of blood, plasma, and red cells in dehydration. J Appl Physiol 37: 247–248. 4850854 10.1152/jappl.1974.37.2.247

[19] Coso JD, Hamouti N, Ortega JF, Fernandez-Elias VE, Mora-Rodriguez R (2011) Relevance of individual characteristics for thermoregulation during exercise in a hot-dry environment. Eur J Appl Physiol 111: 2173–2181. 21305382 10.1007/s00421-011-1847-x

[20] Schiff HB, MacSearraigh ET, Kallmeyer JC (1978) Myoglobinuria, rhabdomyolysis and marathon running. Q J Med 47: 463–472. 751088

[21] Smith JE, Garbutt G, Lopes P, Pedoe DT (2004) Effects of prolonged strenuous exercise (marathon running) on biochemical and haematological markers used in the investigation of patients in the emergency department. Br J Sports Med 38: 292–294. 15155430 10.1136/bjsm.2002.002873 PMC1724837

[22] Clarkson PM, Hubal MJ (2002) Exercise-induced muscle damage in humans. Am J Phys Med Rehabil 81: S52–69. 12409811 10.1097/00002060-200211001-00007

[23] Friden J, Sjostrom M, Ekblom B (1983) Myofibrillar damage following intense eccentric exercise in man. Int J Sports Med 4: 170–176. 6629599 10.1055/s-2008-1026030

[24] Vrijens DM, Rehrer NJ (1999) Sodium-free fluid ingestion decreases plasma sodium during exercise in the heat. J Appl Physiol 86: 1847–1851. 10368348 10.1152/jappl.1999.86.6.1847

[25] Coyle EF, Coggan AR, Hemmert MK, Ivy JL (1986) Muscle glycogen utilization during prolonged strenuous exercise when fed carbohydrate. J Appl Physiol 61: 165–172. 3525502 10.1152/jappl.1986.61.1.165

[26] Millet GY, Tomazin K, Verges S, Vincent C, Bonnefoy R, et al (2011) Neuromuscular consequences of an extreme mountain ultra-marathon. PLoS One 6: e17059. 21364944 10.1371/journal.pone.0017059 PMC3043077

[27] Nybo L, Nielsen B (2001) Hyperthermia and central fatigue during prolonged exercise in humans. J Appl Physiol 91: 1055–1060. 11509498 10.1152/jappl.2001.91.3.1055

[28] Coso J, Estevez E, Mora-Rodriguez R (2008) Caffeine effects on short-term performance during prolonged exercise in the heat. Med Sci Sports Exerc 40: 744–751. 18317369 10.1249/MSS.0b013e3181621336

[29] Saboisky J, Marino FE, Kay D, Cannon J (2003) Exercise heat stress does not reduce central activation to non-exercised human skeletal muscle. Exp Physiol 88: 783–790. 14603378 10.1113/eph8802611

[30] Laursen PB, Suriano R, Quod MJ, Lee H, Abbiss CR, et al.. (2006) Core temperature and hydration status during an Ironman triathlon. Br J Sports Med 40: 320–325; discussion 325. 10.1136/bjsm.2005.022426 PMC2577528 16556786

[31] Abarbanel J, Benet AE, Lask D, Kimche D (1990) Sports hematuria. J Urol 143: 887–890. 2184253 10.1016/s0022-5347(17)40125-x

[32] McInnis MD, Newhouse IJ, von Duvillard SP, Thayer R (1998) The effect of exercise intensity on hematuria in healthy male runners. Eur J Appl Physiol Occup Physiol 79: 99–105. 10052668 10.1007/s004210050480

[33] Gur H, Kucukoglu S, Surmen E, Muftuoglu A (1994) Effects of age, training background and duration of running on abnormal urinary findings after a half-marathon race. Br J Sports Med 28: 61–62. 8044500 10.1136/bjsm.28.1.61 PMC1332164

[34] Reid RI, Hosking DH, Ramsey EW (1987) Haematuria following a marathon run: source and significance. Br J Urol 59: 133–136. 3828707 10.1111/j.1464-410x.1987.tb04803.x

